# Myonuclear Dynamics After Skeletal Muscle Surgical Injury

**DOI:** 10.1096/fj.202602413R

**Published:** 2026-07-14

**Authors:** Micah Goeke, Nathan Serrano, Pieter Jan Koopmans, Kevin A. Murach

**Affiliations:** ^1^ Molecular Muscle Mass Regulation Laboratory, Department of Health, Human Performance, and Recreation University of Arkansas Fayetteville Arkansas USA; ^2^ Cell and Molecular Biology Graduate Program University of Arkansas Fayetteville Arkansas USA

**Keywords:** damage, regeneration, satellite cells, surgery

## Abstract

A hallmark of damaged skeletal muscle fibers is displaced myonuclei that are no longer peripherally positioned. Displaced myonuclei are dogmatically thought to be derived exclusively from muscle stem cell (satellite cell) fusion. Using a surgical resection muscle injury model and in vivo recombination‐independent “resident” (nonsatellite cell‐derived) myonuclear labeling, we detail the prevalence, time course, and origin of displaced myonuclei in response to a nonchemically‐mediated muscle trauma. We found that: (1) nonsatellite cell‐derived (resident) displaced myonuclei emerge 7 days after surgical injury in similar proportion to exogenous (satellite cell‐derived) displaced myonuclei, with a biased prevalence in myosin heavy chain IIB muscle fibers, (2) muscle fibers with multiple (≥ 2) displaced resident myonuclei in cross‐section were an unexpected but noteworthy feature of muscle fibers 7 days after injury, (3) embryonic myosin‐expressing fibers at 7 days postsurgery expectedly contain predominantly satellite cell‐derived displaced myonuclei, but a subset have displaced resident myonuclei, and (4) satellite cell numbers in intact muscle do not increase until 7 days postsurgery. These data may help inform whether to target satellite cell‐initiated processes, myonuclear‐initiated processes, or both to facilitate muscle fiber injury repair. This information could lead to more effective therapeutic strategies for treating muscle trauma.

## Graphical abstract

Using a recombination‐independent doxycycline inducible model of timed nonsatellite cell‐derived myonuclear labeling in adult skeletal muscle, we show that resident myonuclei move and become displaced within intact muscle fibers after muscle surgical injury. Displaced myonuclei may contribute to muscle injury repair, in similar proportion to displaced satellite cell‐derived myonuclei, within a week of injury.

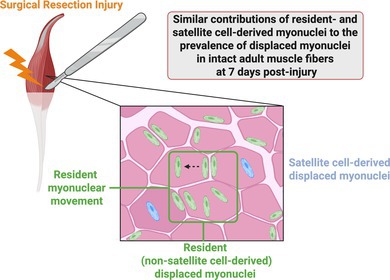

## 1 Introduction

Contemporary knowledge on how skeletal muscle responds to severe trauma has been gathered using models involving myotoxic chemical injections (e.g., barium chloride, cardiotoxin, notexin, etc.) [1, 2], freeze/thermal [2, 3], crush [4], or ischemia–reperfusion injury [5], volumetric muscle loss [6], or eccentric contraction damage [7, 8]. In reality, for humans, the most common form of severe skeletal muscle injury likely occurs during surgery. Muscle is incised to access underlying anatomy or excised (and often discarded) for various reasons. A few classic light and electron microscopy studies reported observations using laceration‐type injuries [9, 10], but in general, detailed studies on how skeletal muscle responds to surgical injury at the cellular and organelle level are scarce [11, 12]. Furthermore, the role of muscle stem cells (satellite cells) has overwhelmingly been the focus of muscle injury repair research. This focus is largely due to the indispensable function of satellite cells in skeletal muscle regeneration [13, 14, 15, 16]. Recent evidence suggests that displaced nonperipheral myonuclei (a hallmark of satellite cell contribution to muscle fibers) [17] can actually be attributed in part to resident (nonsatellite cell‐derived) myonuclear translocation [18, 19, 20]. Resident peripherally‐located myonuclei migrate for the purpose of promoting cell‐autonomous muscle fiber repair processes [20, 21]. Understanding the contributions of resident myonuclei to the injury response is therefore a burgeoning area of interest [21, 22]. The purpose of this investigation was to understand the timing and prevalence of resident versus satellite‐cell derived myonuclei in the context of surgical muscle injury. We used a surgical resection model, a recombination‐independent fluorescent myonuclear labeling mouse model for discriminating resident from satellite cell‐derived myonuclei [18, 19, 23, 24, 25], and displaced myonuclei as a readout of muscle damage repair.

## 2 Study Design

**FIGURE 1:**
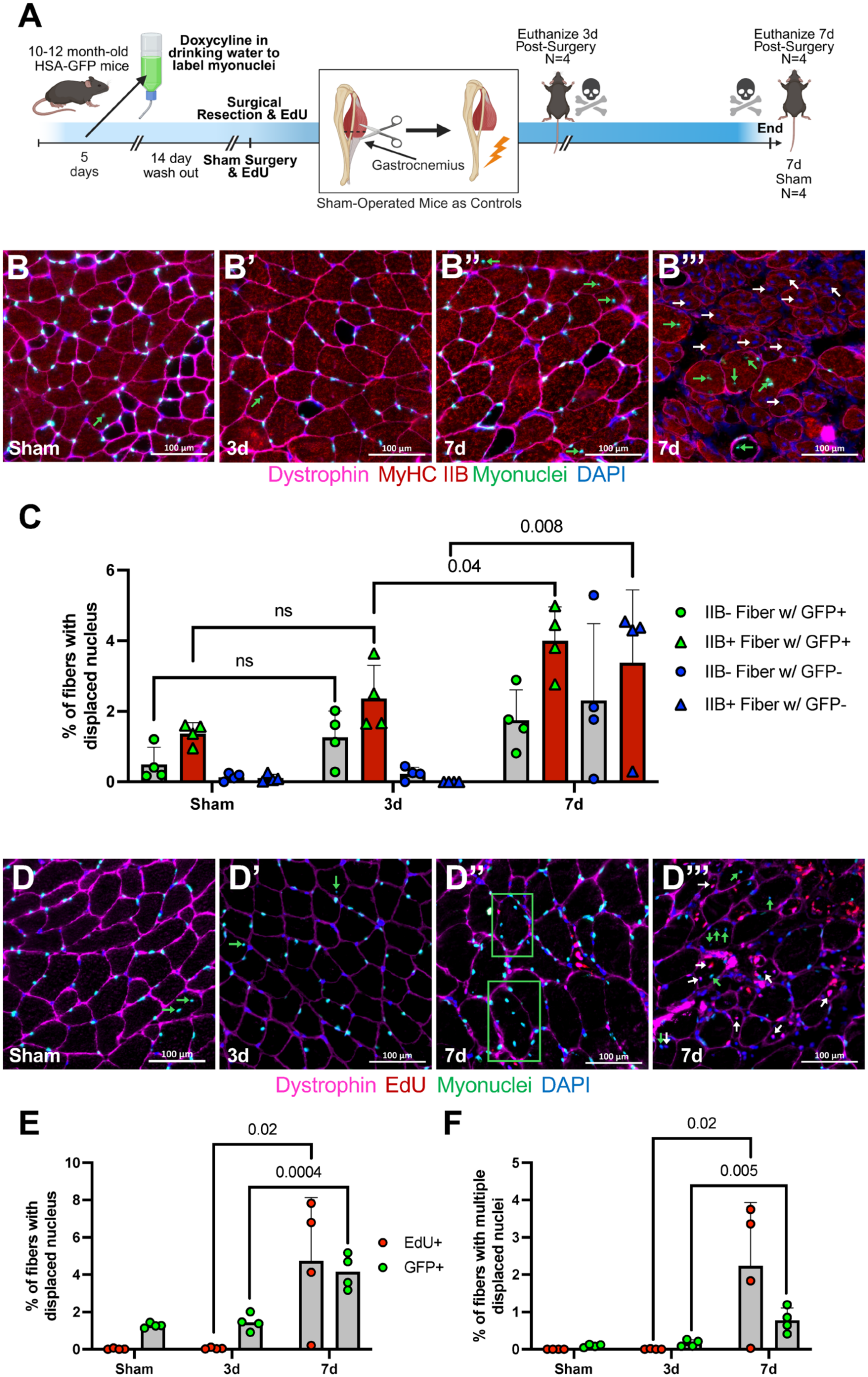
Fiber type‐specific analysis of resident versus non‐resident displaced myonuclei in response to surgical injury. (A) Study design where 10‐month‐old HSA‐GFP mice (M/F = 6/6) were treated with doxycycline to label myonuclei for 5 days followed by a 14‐day wash‐out. The distal ~1/3 of the gastrocnemius‐soleus complex was excised and mice were treated with 5‐ethynyl‐2′‐deoxyuridine (EdU) in drinking water to assess DNA synthesis following surgical resection injury. Resected gastrocnemius muscles were collected at 3 days (*n* = 4; M/F = 2/2) and 7 days (*n* = 4; M/F = 2/2) postsurgery, with sham controls collected at 7 days (*N* = 4; M/F = 2/2). (B–B‴) Show representative images of the gastrocnemius muscle for fiber type‐specific quantification of displaced myonuclei. Images show dystrophin (pink), myosin heavy chain IIB (MyHC IIB, red), GFP‐labeled resident myonuclei (green), and DAPI nuclei (blue). Green arrows = GFP+ resident myonuclei, white arrows = GFP− non‐resident myonuclei. B‴ is a degenerating area with a higher prevalence of displaced myonuclei. (C) Quantification of relative number of IIB+ or IIB− fibers containing one or more displaced myonuclei. Data are presented as mean ± SD and are reported relative to each respective fiber type. (D–D‴) Representative images of the gastrocnemius muscle for DNA synthesis analysis: dystrophin (pink), DAPI (blue), 5‐ethynyl‐2′‐deoxyuridine (EdU, red), with GFP+ resident myonuclei in green. Green arrows = GFP+/EdU− resident myonuclei, white arrows = GFP−/EdU+ exogenous myonuclei. (E) Quantification of relative number of fibers containing multiple GFP+/EdU− or GFP−/EdU+ myonuclei. (F) Quantification of relative number of fibers containing multiple GFP+ or EdU+ nuclei. Significance was established a priori at *p* < 0.05 and assessed via one‐way ANOVA. All data are from whole gastrocnemius muscle cross‐sections, but images represent regions of interest.

Adult HSA‐GFP mice (~10 month old) were given 0.5 mg/mL doxycycline and 2% sucrose for 5 days in drinking water to fluorescently label resident myonuclei. After a 14‐day washout period, mice underwent bilateral muscle resection surgery. The surgery involved removal of the lower ~1/3 of the gastrocnemius/soleus complex and achilles tendon (Figure 1A). Mice returned to normal ambulation (relying on the plantaris muscle for plantarflexion) within ~24 h [18]; the gastrocnemius muscles from Serrano et al. were analyzed here [18]. Resected muscles were collected 3 days (*n* = 4; M/F = 2/2) or 7 days after surgery (*n* = 4; M/F = 2/2), and sham operated mice (no resection and time‐matched to the 7‐day cohort, *n* = 4; M/F = 2/2) were controls. At the time of surgery, mice were given EdU in drinking water according to Serrano et al. [18]; this approach results in appreciable DNA labeling in our hands [18]. Resected muscles (whole gastrocnemius) were prepared for histology according to our standard procedures [18] and analyzed for displaced myonuclei: “resident” GFP+/DAPI+ or “exogenous” (i.e., satellite cell‐derived) GFP−/DAPI+ and not abutting the dystrophin border (called GFP+ or GFP− from here onward), quantified according to myosin heavy chain 2B expression (MyHC IIB+ or IIB−, with IIB+ being pure IIB and/or IIB/IIX, and IIB− likely being IIA and/or IIX fibers) or embryonic myosin heavy chain fiber type expression (eMyHC+ or eMyHC−). The muscles were analyzed a few millimeters away from the injury site. Displaced myonuclei were also quantified as EdU+ or EdU−. Satellite cells per fiber were identified as PAX7+/DAPI+ and within the muscle fiber laminin border [26, 27].

## 3 Three‐Day Postsurgical Myonuclear Response

GFP+ (resident) displaced myonuclei were not significantly more abundant relative to sham in MyHC IIB− or IIB+ muscle fibers (Figure 1B,C); however, IIB+ fibers were beginning to feature modestly more displaced resident myonuclei (1.4% vs. 2.4%, *p* = 0.23). There was greater variability in displaced resident myonuclei in both fiber types with surgery relative to sham; perhaps a larger sample size may have revealed significance, or a more comprehensive study involving longitudinal muscle sections and/or multiple analysis sites may be required to identify the possible source(s) of variability at this time point. GFP− (presumably satellite cell‐derived) displaced myonuclei were not more abundant in MyHC IIB+ or IIB− muscle fibers 3 days after surgical resection (~0% in both fiber types). In response to mechanical overload (MOV) of the plantaris muscle, IIB− fibers had significantly more displaced resident myonuclei versus sham after 3 days in our hands [18]. Mechanical tension may, in part, drive early myonuclear translocation in IIB− muscle fibers, whereas damage alone may not. Relative to the plantaris, though, IIB− (IIA and/or IIX) fibers are less prevalent in the gastrocnemius. This relative scarcity may also contribute to the conclusions from our analysis.

## 4 Seven‐Day Post‐Surgical Myonuclear Response

Seven days after surgical resection, IIB− fibers did not have significantly more GFP+ or GFP− displaced myonuclei; however, there was again more variability relative to sham (Figure 1C). IIB+ muscle fibers had more displaced GFP+ and GFP− displaced myonuclei 7 days after injury relative to sham (IIB+/GFP+: *p* = 0.002; IIB+/GFP−: *p* = 0.009), and more versus 3 days after surgery (IIB+/GFP+: *p* = 0.04; IIB+/GFP−: *p* = 0.008) (Figure 1B,C). In total, IIB+ fibers had ~4% of fibers with GFP+ and ~3.5% GFP− displaced myonuclei 7 days after injury (~7.5% of total fibers). This overall prevalence exceeds what is found after 7 days of MOV (< 4% of total fibers with displaced myonuclei) [18]. There were some regional differences where intact myofibers predominantly contained GFP+ displaced myonuclei, whereas regions undergoing degeneration (smaller fibers with large interstitial spacing) were dominated by GFP− displaced nuclei (Figure 1B″ and B‴, respectively). One sample had an unexpected phenotype where GFP+ displaced myonuclei were prevalent 7 days after injury but GFP− were not (Figure 1C). Our analysis of this muscle may have been more distant from the injury site versus the others, or perhaps a portion of resected muscle had actually remained intact in this sample. Nevertheless, we elected to retain this sample in the analysis. The timing of events—where GFP+ displaced myonuclei may begin emerging early after injury and become more prevalent over time—agrees with classic work where myofiber membrane sealing (cell‐autonomous process) precedes satellite cell fusion (noncell‐autonomous process) after injury [28], and reinforces speculation where myonuclear migration explains early myonuclear accumulation at the damaged ends of intact myofibers [9, 28].

**FIGURE 2:**
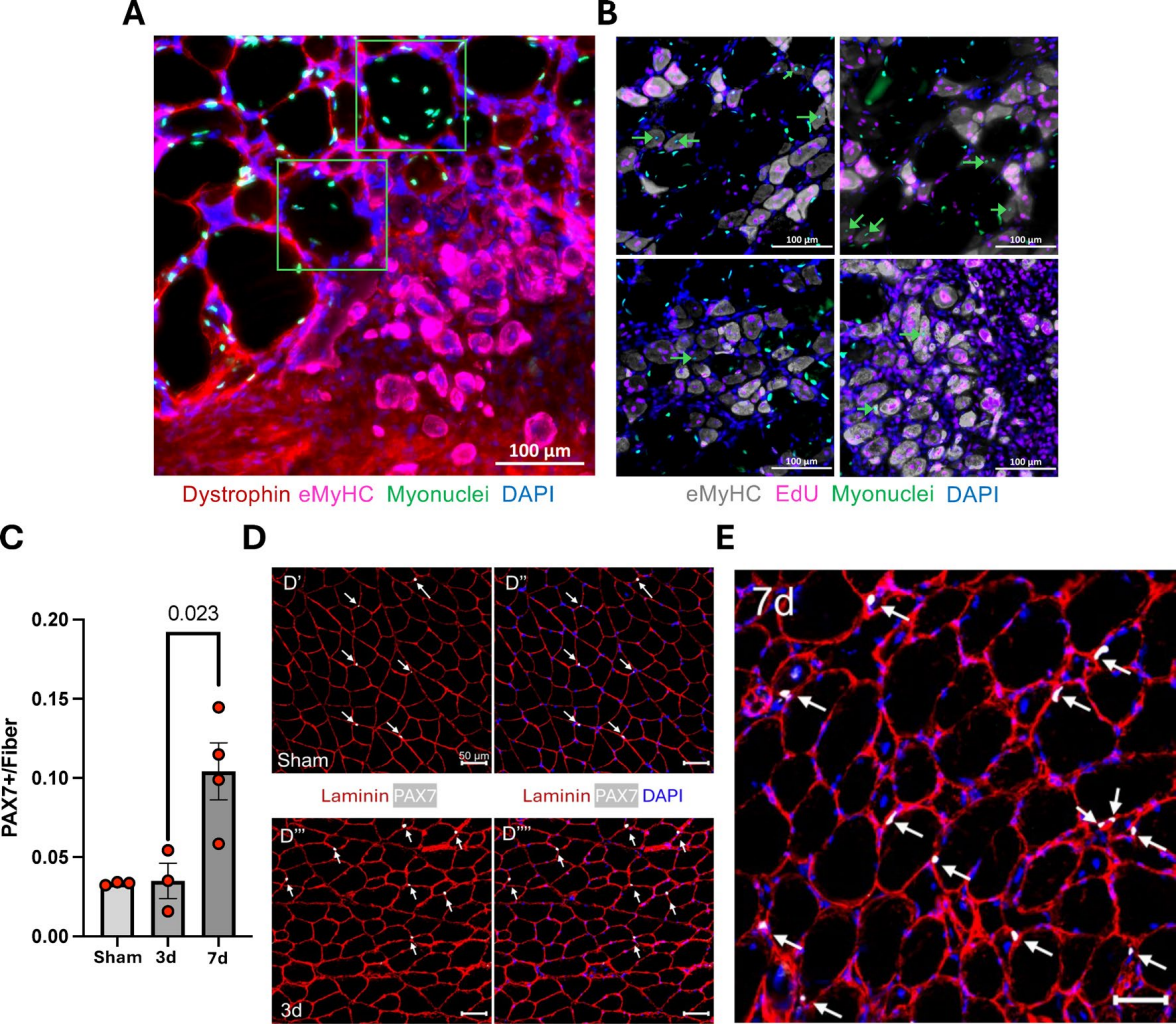
Displaced myonuclei in eMyHC‐expressing muscle fibers and muscle fibers with multiple displaced resident myonuclei after surgical injury. (A) Z‐stack image from 30 μm section of 7 day gastrocnemius showing regional eMyHC abundance and prevalence of several displaced resident myonuclei within a single fiber. (B) Representative images of emybronic myosin positive fibers with displaced resident myonuclei. Green arrows = GFP+ resident myonuclei. Degenerating areas were selected to demonstrate the prevalence of displaced myonuclei in those regions. (C) Quantification of number of satellite cells at each timepoint. Data are presented as mean ± SD and are reported relative to the number of fibers. (D) Representative images of satellite cells in the gastrocnemius muscle at sham (D and D′) and 3 day timepoints (D″ and D‴). Laminin (red), PAX7 (white), and DAPI (nuclei, blue). (E) Representative image of satellite cells (white arrow) in the gastrocnemius muscle at 7 day timepoint. Scale bar is 50 μm in panels (D) and (E). All data are from whole gastrocnemius muscle cross‐sections, but images represent regions of interest.

## 5 DNA Synthesis Analysis of Displaced Myonuclei Three and Seven days After Surgery

Nearly all displaced GFP+ myonuclei in injured muscle were EdU−, and displaced GFP− were EdU+; the latter were most likely derived from satellite cells that had proliferated and fused to the muscle fiber. Consistent with the GFP+/GFP− analysis presented above, the abundance of displaced GFP+ and EdU+ myonuclei in intact muscle fibers was not significantly higher until 7 days after surgical injury (EdU+: 3 day vs. 7 day: *p* = 0.02; GFP+: 3 day vs. 7 day: *p* = 0.0004) (Figure 1D‴ and 1E). A unique and striking feature of surgical injury compared to mechanical overload [18] with respect to displaced myonuclei was the relative preponderance of muscle fibers with multiple (≥ 2) displaced myonuclei in cross‐section specifically after surgery (see Figure 1D″–D‴). There were more individual fibers with multiple displaced myonuclei that were EdU+ (likely regenerating, up to 120 fibers per muscle cross‐section, ~2.5% of all fibers); however, up to 30 fibers per muscle (< 1% of all fibers) had multiple GFP+ displaced resident myonuclei (likely damaged but intact fibers) 7 days after injury (EdU+: 3 day vs. 7 day: *p* = 0.02; GFP+: 3 day vs. 7 day: *p* = 0.005) (Figures 1D–D‴ and 1F). We obtained thicker muscle sections (30 μm) to better visualize these fibers and myonuclei (Figure 2A). Unlike the organized, linear, and centralized “myonuclear chains” that often occur during regeneration [17, 29], it appears that these GFP+ resident myonuclei were scattered throughout the inside of the muscle fiber along the same cross‐sectional plane. Few fibers had multiple displaced GFP+ myonuclei in the sham condition (Figure 1F). Our observations provide evidence that resident myonuclei can mobilize *en masse* in response to surgical damage in intact adult muscle fibers.

## 6 eMyHC+ Muscle Fibers With GFP+ Displaced Myonuclei

Regenerating muscle fibers expressing eMyHC and GFP− and/or EdU+ displaced myonuclei would be expected to emerge in response to severe injury since regeneration and eMyHC expression are driven by satellite cells [13]. We nevertheless evaluated eMyHC+ muscle fibers with GFP+ displaced myonuclei. These “double positive” fibers were rare, comprising a small proportion of all eMyHC+ fibers (Figure 2B). Despite their rarity, resident displaced myonuclei in eMyHC+ fibers may be an unrecognized but potentially meaningful feature of muscle fiber degeneration/regeneration since we have observed these fibers before following unacccustomed muscle activity [19].

## 7 Satellite Cell Abundance After Surgical Resection Injury

With our surgical resection approach, the achilles tendon is excised along with the muscle which leads to the removal of muscle loading on the resected muscle. Surgical resection is therefore a damage model simultaneous with an atrophy stimulus (i.e., deloading and possible denervation). How satellite cells react to such a stimulus is not well‐described. Muscle damage typically causes satellite cell proliferation [8] whereas deloading either does not affect satellite cell number [30] or reduces it [31, 32]. We therefore evaluated the abundance of PAX7+ satellite cells to further characterize the resection model in intact muscle fibers where clear laminin borders were discernible. At 3 days, satellite cell abundance in intact regions of muscle was not changed (Figure 2C,D–D′) but at 7 days, satellite cells were ~3‐fold more abundant (sham vs. 7 days, *p* = 0.021; 3 days vs. 7 days, *p* = 0.023) (Figure 2D,E). In some regions undergoing active regeneration, satellite cells were very abundant but could not be easily quantified due to disrupted laminin architecture.

## 8 Conclusions

Displaced resident myonuclei appear in response to unaccustomed physical activity in the soleus muscle [19] and mechanical overload of the plantaris muscle [18], but most prominently with surgical resection in the gastrocnemius of adult muscle shown here. Although our sample size is limited, it was sufficient to identify approximately twice the prevalence of displaced resident myonuclei with surgical injury versus mechanical loading in these same animals at 7 days [18]. In the current study, we cannot confidently say whether every muscle fiber in cross‐section was damaged directly. This uncertainty is in part due to the site of injury and the pennation angles of the medial and lateral gastrocnemius muscles. We also cannot say definitively whether displaced resident myonuclei are the direct consequence of damage since displaced myonuclei can be a feature of muscle fibers in response to less catastrophic stressors such as minimally injurious voluntary exercise [33], muscle aging independent from severe injury [34, 35], and muscle loading in the absence of overt degeneration/regeneration [36]. Displaced myonuclei are also characteristic of denervation as well as certain nondegenerative myopathies [37] and cancer cachexia [38, 39]. On balance, it is intuitive that displaced resident myonuclei are the result of damage, and every fiber may have had some amount of displaced resident myonuclei but we did not capture them due to the site we chose to analyze as well as the narrow thickness of our muscle sections (7–30 μm). Nevertheless, in intact (eMyHC−) muscle fibers not undergoing discernible regeneration, displaced resident myonuclei were a prominent feature that is either directly related to damage or indirectly related to inflammation, denervation, and/or deloading. In our hands, resident myonuclear movement is a conserved feature across muscle types and stressors.

## 9 Methods

All experimental conditions were the same as in Serrano et al. [18]. Fiber type (including eMyHC), DAPI, Dystrophin, EdU, and Pax7 histology were performed according to our previously published protocols [18, 19, 26, 27, 33, 36]. Muscle fiber counts on whole muscle cross‐sections were assessed using MyoVision semiautomated analysis software [32]. Displaced myonuclei counts were analyzed manually. One‐way ANOVAs were performed independently on relative displaced myonuclear counts according to the individual populations over time (Sham, 3d, 7d): IIB+/GFP+, IIB+/GFP−, IIB−/GFP+ and IIB−/GFP− nuclei. For proliferative status, populations were: GFP+/EdU−, GFP−/EdU+, and fibers containing multiple (≥ 2) displaced nuclei. Tukey's post hoc corrections were applied to all ANOVAs. Statistical significance was set at *p* ≤ 0.05. All figures were created using GraphPad Prism and the graphical abstract was made using BioRender.

## Author Contributions

N.S., P.J.K., and K.A.M. conceived of the study analysis approaches. M.G., N.S., and P.J.K. analyzed data. K.A.M. wrote the manuscript with input from M.G., N.S., and P.J.K. M.G., N.S., and P.J.K. generated figures. M.G., N.S., P.J.K., and K.A.M. managed and/or performed experiments. K.A.M. provided resources, oversight, and/or intellectual contributions. All authors provided feedback and final approval of the manuscript.

## Conflicts of Interest

The authors declare no conflicts of interest.

## Data Availability

All histology data that were quantified are presented in the manuscript.
